# A Novel Technique of Total Laparoscopic Hysterectomy for Routine Use: Evaluation of 140 Cases

**Published:** 2008-03

**Authors:** S. P. Puntambekar, G. N. Wagh, S. S. Puntambekar, R. M. Sathe, M. A. Kulkarni, M. A. Kashyap, A.M. Patil, Meinhold-Heerlein Ivo

**Affiliations:** 1 *Galaxy Laparoscopy Institute, Pune, India;*; 2 *Department of Gynecology and Obstetrics, University Hospital of Schleswig-Holstein-Campus Kiel, Kiel, Germany*

**Keywords:** hysterectomy, laparoscopy, uterine pathology

## Abstract

Hysterectomy is one of the most commonly performed gynecological procedures. Although the first laparoscopic hysterectomy was performed in 1989, this technique accounts for only a few of all hysterectomies performed today. To assess the safety of total laparoscopic hysterectomy through a novel technique that we have evolved, a retrospective analysis of 140 patients with benign uterine pathologies operated at our institute between 2004 and 2007 was performed. All patients underwent total laparoscopic hysterectomy (TLH) using a simple technique. The highlight of this technique was the omission of any vaginal manipulator. The mean operation time was 88.75 ± 52.72 minutes, the mean blood loss 53.80 ± 35.94 ml and the mean hospital stay 2.21 ± 1.12 days. No conversion to open surgery was necessary. Iatrogenic complications were bowel injury (n=1) and vaginal tears (n=3) and were managed laparoscopically. The new method of TLH proved to be reproducible and safe with decreased morbidity and operation time. This can be attributed to the performance of the same standardized steps each time. Our technique provides a safe procedure suitable for routine use in gynecological surgery.

## INTRODUCTION

Hysterectomy is the commonest gynecological procedure performed and increasingly so in the younger age group (1). The first laparoscopic hysterectomy was performed and published in 1989 (2), but this surgical technique started gaining widespread acceptance from 1991 onwards (3, 4, 5).

Since the introduction of laparoscopic hysterectomy, several modifications have been described. These are laparoscopic assisted vaginal hysterectomy (LAVH) (2), laparoscopic assisted supracervical hysterectomy (LSCH) (6) and total laparoscopic hysterectomy (TLH) (7). Although the progress in laparoscopic surgery made over the past few years has proven that total laparoscopic hysterectomy is feasible and reproducible (8, 9), it has not even partly replaced the open technique due to the increased number of complications associated with it. Therefore, a safe technique that can be used routinely with basic instruments and standardized steps may contribute to increase the acceptance of TLH.

Besides being safe, the method used should maintain the length of the vagina, vault supports as well as the integrity of the pelvic floor. If the rate of complications can be reduced by introduction of a simple and safe method, TLH may supersede all other techniques presently used.

The main purpose of this article is to describe a safe and an easy, reproducible technique with standardized steps evolved in our institution.

## MATERIALS AND METHODS

### Analysis of patient’s data

A retrospective evaluation was done, of all patients (n=140) who underwent TLH for benign uterine pathologies from January 2004 to October 2007 at the Galaxy Laparoscopy Institute, Pune, India.

The following parameters were determined in this observational study: Age, BMI, indication for surgery, past medical and surgical history, intra-operative observations, postoperative complications and duration of hospital stay.

The patients undergoing hysterectomy were in the age group of 34-76 years, the mean age being 45.32 ± 6.51 years. The average body mass index was 25 with 18 patients having body mass index >30. 61 patients had a significant past medical history of hypertension (n=30), diabetes (n=14), hypothyroidism (n=4), chronic anemia (n=12) and tuberculosis (n=1). 129 patients had a history of previous abdominal surgery like minilap tubal ligation (n=76), appendicectomy (n=7), cholecystectomy (n=2) and lower segment caesarean section (n=28). Diagnostic laparoscopy was done in 13 patients and 3 patients had undergone laparotomy, for abdominal lump, adhesiolysis for sub acute intestinal obstruction and for an unknown indication, respectively. The indications for hysterectomy are enumerated in Table 1.

**Table 1:** Indications for hysterectomy

Indications	No. of patients

Dysfunctional uterine bleeding (not responding to medical treatment)	66
Large fibroid	27
Pelvic inflammatory disease	9
Endometriosis (including Adenomyosis)	20
Cervical polyp	5
Cervical dysplasia	3
Adnexal Mass	10

### Preoperative workup

Patients were thoroughly evaluated with relevant preoperative work up for hysterectomy and assessment of anesthesia risks. Pap smear, endometrial sampling and ultrasound investigation ruled out malignancy. No cases were excluded on the basis of uterine size (largest size being 32 weeks) and mobility. Preoperative preparation included a written informed consent, counseling with respect to oophorectomy, need for conversion to laparotomy and complications. Bowel preparation was done the night prior with polyethylene glycol.

### Patient’s Position

**Figure 1:**
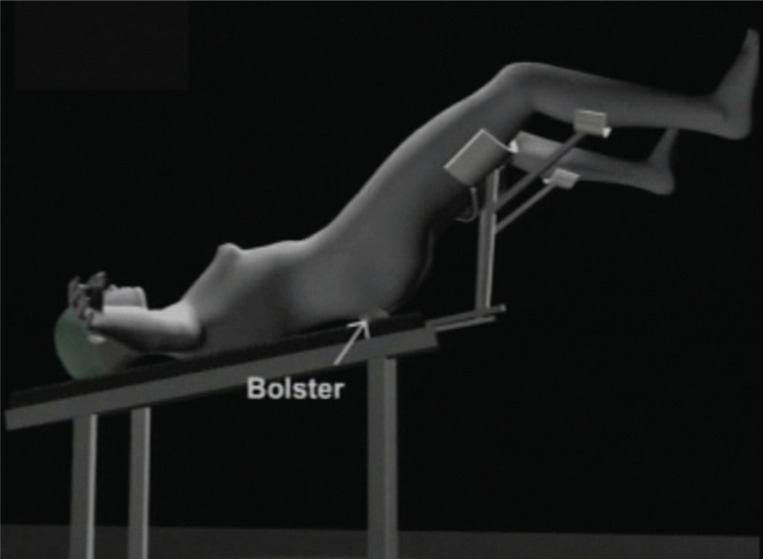
Patient position. The modified Lloyd Davis position with a bolster under the buttocks is shown.

After application of a combination of spinal and general anesthesia, the patient was placed in a modified Lloyd-Davis position with a bolster under the buttocks at the level of the anterior superior iliac spines (Figure 1). This offers elevation of the pelvis and results in a drop of the intestines cephalad, offering a comfortable access to the pelvis. A gauze piece was kept in the vagina to prevent loss of pneumoperitoneum after colpotomy.

### Position of the surgeons

**Figure 2:**
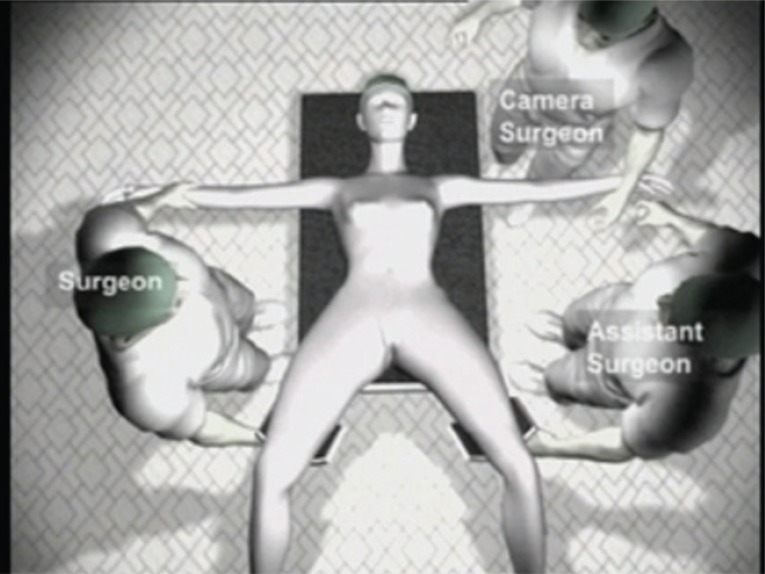
Position of surgeons and assistants. The operating surgeon stands on the right side of the patient. The assistant surgeon and the camera person stand on the left.

The operating surgeon stood on the right side of the patient. The assisting surgeon and the camera-person stood on the left. The assisting staff nurse stood on the right side of the surgeon. Two monitors were placed near the foot end of the patient for the surgeon and the assistants respectively (Figure 2).

### Port positions

**Figure 3:**
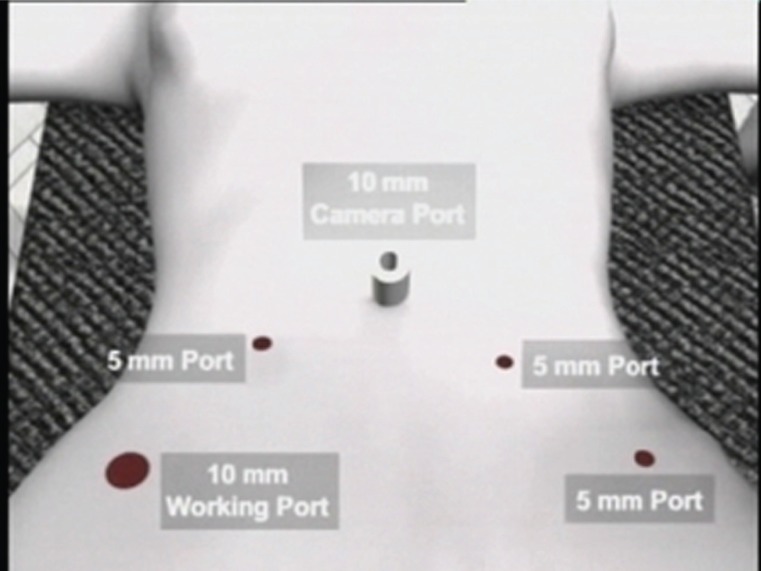
Port positions. The five standard pelvic ports introduced were as follows: **1)** A 10 mm port at the umbilicus for the telescope, camera, light source and the CO_2_; **2)** A 10 mm port at the right Mc Burney’s point for the surgeon’s operating port; **3)** A 5 mm port at the right mid-clavicular line at the level of the umbilicus for the surgeon’s manipulating port; **4)** A 5 mm port as a mirror image of port no 2) for the myoma screw; **5)** A 5 mm port as a mirror image of port no 3) for bladder and bowel retraction.

We used the open technique of primary port insertion under vision by accessing the umbilical tube. The port positions are shown in Figure 3. In cases of large uteri and suspected adhesions, the Palmer’s point access with the Verress needle was made.

### Procedure of operation

After inserting a 10 mm ‘0’ degree telescope, the pelvis and upper abdomen was visualized to thoroughly assess any kind of pathology. The small intestines were packed in the right iliac fossa. A myoma screw was inserted into the uterine fundus and the uterus retracted cephalad, and to the left. The right round ligament, ovarian ligament and the fallopian tube were coagulated and cut. The anterior leaf of the broad ligament was opened and an anterior ‘U’incision taken through the uterovesical fold. The urinary bladder was dissected over the cervix by sharp and blunt dissection. A gauze piece kept at this point helped to define the bladder extent by acting as a contrast and also helped to control small capillary bleeding. The uterus was then retracted to the right. The left fallopian tube, ovarian and round ligament complex was coagulated, cut and the anterior U cut completed. The posterior peritoneum behind the uterine vessels was kept intact at this stage. The left hand working forceps was placed at the level of the uterosacral ligaments to lift the cervix anteriorly and away from the sigmoid colon. The left uterine vessels were identified, coagulated and cut. Once the uterine artery was cut, all the clamps were applied parallel to the cervix and medial to the uterine artery similar to sliding the clamps over the cervix as done in open surgery. A similar dissection was done on the right side and the right uterine arteries were coagulated and cut. With continuous traction on the uterus the posterior peritoneum and the parametrial tissue on the lateral aspect of the cervix was coagulated and cut. The dissection was always kept above the level of the uterosacral attachment. The parametrial tissue at the vaginal angle was gradually separated with coagulation. Colpotomy was performed from the right vaginal angle towards the left, always staying very close to the cervix, as if the cervix was circumcised off its uppermost vaginal attachment. Colpotomy was performed by using bipolar forceps and scissors. Oophorectomy when indicated was performed at this stage by coagulating and cutting the infundibulopelvic ligaments. The ovaries and the uterus were extirpated per vaginum. The loss of pneumoperitoneum after removal of the specimen was prevented by re-inserting a gauze piece in the vagina. When the uterus was large, it was morcellated and removed through the right lower port. The vagina was sutured with 2, 0 vicryl continuous locking intra-corporeal suturing technique, either ipsilateral or contralateral.

### Energy sources

Majority of the time, TLH was performed with bipolar cautery and scissors. But we have also used a combination of bipolar, Valleylab Vessel Sealing Device (Tyco, Boulder, Colorado, USA), Plasmakinetic vessel sealing system (PK, Gyrus International Ltd., Berkshire, UK) and the Harmonic ACE™ (Ethicon Endosurgery Inc. Aurangabad, India). We did not use monopolar current. The technique remained the same irrespective of the energy source used.

In difficult cases like in patients having large myomas and endometriosis, ureteric stents were placed preoperatively. In such cases, identification and dissection of the ureters was undertaken as the first step. During difficult posterior dissection, the dictum followed was that ‘fat always belongs to the rectum’, and so the dissection was kept anterior to the fat. Similarly during bladder dissection, the rule followed was that ‘fat always belongs to the bladder’, and therefore the dissection was done posterior to this fat.

## RESULTS

140 patients underwent total laparoscopic hysterectomy at our institute during the period of January 2004 to October 2007.

As indicated in Table 2, the average time required for surgery was 88.75 ± 52.72 minutes. The average blood loss was 53.80 ± 35.94 ml. There were no anesthetic complications and conversion to laparotomy was not required in any of the cases. Adhesions were found in 29 cases and a bicornuate uterus was seen in one. The average vaginal margin on the specimen was less than 5 mm.

**Table 2:** Parameters analyzed

Parameters	Mean

Age	45.32+/- 6.51 years
Time required for surgery	88.75+/- 52.72 min
Blood loss	53.80+/- 35.94 ml
Hospital stay	2.21+/- 1.12 days

2 patients with history of previous caesarean section had cystotomy during bladder dissection which was recognized intra-operatively and sutured with 2.0 vicryl in a single layer by continuous locking intra-corporeal sutures. 2 patients had a vaginal wall tear during delivery of the uterus per vaginum, which was immediately sutured. 1 patient had sigmoid colon perforation because of unanticipated maneuver by an assisting surgeon, which was recognized and sutured intra-operatively. One patient developed sepsis due to lower respiratory tract infection, which was treated with higher antibiotics (Table 3). Bowel sounds appeared within 6 to 8 hours. 2 patients developed paralytic ileus and were managed conservatively. The complications are summarized in Table 3. Patients were ambulated after 24 hours. 10 patients were discharged after 24 hours, 109 after 48 hours and 10 after 72 hours. 6 patients were discharged after 4 days and 3 patients after 5 days. Only 1 patient who developed postoperative sepsis had a long hospital stay of 17 days (Table 2).

**Table 3:** Complications

a) Intra-operative complications			
Complication	Number of patients	Management	Post-operative course

Cystotomy	2	Sutured intra-operatively. Catheter for 21 days.	Uneventful. Patient discharged on Day 3.
Bowel perforation	1	Sutured intra-operatively. Nil by mouth for 3 days. Higher antibiotics.	Uneventful. Discharged on Day 5.
Vaginal wall tear	2	Sutured intra-operatively	Discharged on Day 3.

**b) Postoperative complications**			
**Complication**	**Number of patients**	**Management**	**Course**

Paralytic ileus	2	Conservative	Uneventful. Discharged on Day 4
Sepsis	1	Higher antibiotics	Satisfactory. Discharged on Day 17

## DISCUSSION

Various techniques of TLH have been described in the literature. Laparoscopy is a safe route provided the surgeons are well trained, because then the rate of complications is not higher than that observed with laparotomy or by the vaginal route (10, 11, 12, 13, 14). The American College of Obstetricians and Gynecologists guidelines state that the route of hysterectomy should depend on the patient’s anatomy and surgeon’s experience (15). Many laparoscopic surgeons all over the world still prefer LAVH over TLH (16).

Another point of debate is the preference of Supracervical laparoscopic hysterectomy (SLH) to TLH, the advantage of SLH being conservation of the pelvic floor integrity and sexual pleasure (17), which is still controversial (18, 19). However in our country, there are no supporters to this view due to high incidence of cancer cervix and the lack of good screening programs and patients’ follow up. In a recent paper, Okaro *et al* (20) reported their long term experience with 70 patients who underwent SLH. The mean follow up time was 66 months. In 17 women (24.3%) pathological symptoms were reported: pelvic pain and dyspareunia in 13 (18.6%), vaginal bleeding in eight (11.4%), and vaginal discharge in another three (4.3%) patients. All of these 17 women required further surgery with removal of the cervical stump in 16 (22.3%), which is a more difficult and morbid procedure.

Years after the first case of TLH was published (2); this operative procedure is performed in relatively few centers worldwide. The reasons for this restriction can be unavailability of a formal curriculum, lack of standardization of procedures and training (21). Therefore, a proper training program with a standardized procedure is necessary for the education of the resident and fellow doctors to qualify them for coping with the possible difficulties encountered during this surgery. In our opinion, through the use of standardized steps, TLH can become an easy procedure which can be mastered by many.

The salient steps of our technique are (a) use of a combination of regional and general anaesthesia, (b) ergonomic port, patient and surgeon positioning, (c) the use of a myoma screw, which gives cephalad traction of the uterus, instead of any vaginal manipulator, (d) sharp dissection of the bladder, (e) applying the bipolar forceps medial to the uterine stump, in the same manner as applying clamps in abdominal hysterectomy, (f) keeping the left hand working forceps at the level of the uterosacral ligaments to lift the cervix anteriorly (g) remaining above the uterosacral ligaments.

A high morbidity rate is reported in the literature for laparoscopic hysterectomies. If we analyze these studies, we find that complications usually arise during the learning curve of the new procedure (22). A recent publication from Finland analyzing prospectively 10110 hysterectomies performed nationwide revealed that with increasing experience of more than 30 consecutive surgeries performed by the surgeon, the number of complications was significantly decreased (23). Our technique of TLH requires less operative time, has minimal blood loss and decreased morbidity. This can be attributed to the performance of the same standardized steps every time in the surgeries. This makes the surgeon well versed with the technique and decreases the rate of complications.

With the development of technology, uterine size remains a relative contraindication. Other relative contraindications could be related to any technical problems in abdominal entry and the body mass index (24, 25). Abdominal entry would be difficult in women with repeated cesarean sections or previous laparotomy, especially with midline incision where the chances of organ adhesion are up to 50%. In obese patients (body mass index more than 30), the rate for major intra-operative complications is increased compared with those reported for women with a lower BMI. The cited reference mentions the difficulties in obese women which are related to anesthesia and the creation of pneumoperitoneum.

For bowel preparation we use peglec (poly ethylene glycol) a night prior to surgery instead of enema. This prevents gaseous distension of bowel. We prefer to use a combination of general and regional anesthesia in all patients. Regional anaesthesia causes contraction of the bowel and hypotension due to sympathetic blockade, and thus ensures a clear operative field. It also decreases the requirement of drugs in anaesthesia. Addition of general anaesthesia helps in controlled ventilation and maintenance of the vital parameters by washing out the CO_2_. It also prevents patient discomfort during surgery, which may be due to steep head low and pneumoperitoneum. We give a modified Lloyd Davis position with a bolster under the buttocks instead of the lithotomy position. The open entry method by cutting the umbilical tube is a safe and easy method of primary port insertion especially in obese patients. By not cutting the posterior peritoneum and by keeping the left working forceps at the level of the uterosacral ligaments while coagulating the uterine vessels, damage to the bowel is prevented and the lateral energy spread can be limited. Staying above the uterosacral ligaments prevents damage to the ureters, maintains a good vaginal length and aids in preventing a vault prolapse. For simplification of the procedure, oophorectomy is done last as the ovaries obstruct the operative field if the infundibulopelvic ligaments are cut before completing the steps of hysterectomy. We follow definite protocols in anticipation of problems in difficult cases like endometriosis and large fibroids, where we identify and dissect the ureters and clip the major vessels before beginning with the standard steps of the surgery.

Our technique of TLH has proved to be a safe and feasible alternative to open hysterectomy. We attribute the safety of the procedure to the evolution of a standard technique involving simple steps which are performed repetitively. This technique can be mastered and duplicated easily. It reduces the morbidity associated with hysterectomy and has the potential to replace hysterectomy by laparotomy in benign disease.

## CONFLICT OF INTEREST

The authors declare that no conflicting interests exist.
